# Analysis of the Mediterranean fruit fly [*Ceratitis capitata* (Wiedemann)] spatio-temporal distribution in relation to sex and female mating status for precision IPM

**DOI:** 10.1371/journal.pone.0195097

**Published:** 2018-04-04

**Authors:** Andrea Sciarretta, Maria Rosaria Tabilio, Elena Lampazzi, Claudio Ceccaroli, Marco Colacci, Pasquale Trematerra

**Affiliations:** 1 Department of Agriculture, Environmental and Food Sciences, University of Molise, Campobasso, Italy; 2 CREA, Consiglio per la ricerca in agricoltura e l’analisi dell’economia agraria, Research Centre for Olive, Citrus and Tree Fruit, Rome, Italy; 3 ENEA, Italian National Agency for New Technologies, Energy and Sustainable Economic Development, Casaccia Research Center, Rome, Italy; University of Thessaly School of Agricultural Sciences, GREECE

## Abstract

The Mediterranean fruit fly (medfly), *Ceratitis capitata* (Wiedemann), is a key pest of fruit crops in many tropical, subtropical and mild temperate areas worldwide. The economic importance of this fruit fly is increasing due to its invasion of new geographical areas. Efficient control and eradication efforts require adequate information regarding *C*. *capitata* adults in relation to environmental and physiological cues. This would allow effective characterisation of the population spatio-temporal dynamic of the *C*. *capitata* population at both the orchard level and the area-wide landscape.

The aim of this study was to analyse population patterns of adult medflies caught using two trapping systems in a peach orchard located in central Italy. They were differentiated by adult sex (males or females) and mating status of females (unmated or mated females) to determine the spatio-temporal dynamic and evaluate the effect of cultivar and chemical treatments on trap catches. Female mating status was assessed by spermathecal dissection and a blind test was carried out to evaluate the reliability of the technique. Geostatistical methods, variogram and kriging, were used to produce distributional maps. Results showed a strong correlation between the distribution of males and unmated females, whereas males versus mated females and unmated females versus mated females showed a lower correlation. Both cultivar and chemical treatments had significant effects on trap catches, showing associations with sex and female mating status. Medfly adults showed aggregated distributions in the experimental field, but hot spots locations varied. The spatial pattern of unmated females reflected that of males, whereas mated females were largely distributed around ripening or ripe fruit. The results give relevant insights into pest management. Mated females may be distributed differently to unmated females and the identification of male hot spots through monitoring would allow localisation of virgin female populations. Based on our results, a more precise IPM strategy, coupled with effective sanitation practices, could represent a more effective approach to medfly control.

## Introduction

The Mediterranean fruit fly (medfly), *Ceratitis capitata* (Wiedemann) (Diptera: Thephritidae), is considered one of the world’s most destructive pests. Medfly is able to infest the fruits of over 300 species of plants, adapt to a wide range of climatic zones and have an elevated invasive potential [1–7]. Medfly can attack commercially important fruit, thus causing considerable economic damages, estimated to be more than 2 billion dollars annually [8].

*Ceratitis capitata* originated in the sub-Saharan Africa and can be found in many tropical, subtropical and mild temperate regions worldwide [9,10]. There are quarantine restrictions on the import of products from medfly-infested countries [11,12] and costly detection and eradication programmes are in place to avoid the establishment in new regions [13,14]. In addition, global warming may allow the expansion of medfly geographical distribution to higher latitudes. This is an ongoing phenomenon observed, for example, in northern Italy [15]. A variety of climate matching and ecologically niche models have been used to assess the potential geographic distribution of the medfly in different parts of the world [16–20].

Traditionally, medfly has been controlled using insecticides (organophosphates and pyrethroids). To limit the negative effect of cover treatment in the orchard ecosystem, pesticides are often applied selectively. Baits, made up of an attractive substance and an insecticide such as deltamethrin, spinosad or lufenuron, are sprayed over part of the tree canopy or localised in specific devices [21–24]. Other control approaches include mass trapping [25–27], the sterile insect technique (SIT) [14,23,28–30], and biological control [31–32]. One of the most effective control techniques is to wrap the fruit in a paper bag, or in the case of long, thin fruit, a polyethylene sleeve [10].

In all of these cases, pest control techniques target adults, as eggs deposited in the fruit are hard to kill. Therefore, efficient medfly control can be achieved by defining and delimiting the spatial and temporal distribution of adult fly populations. This information can be largely obtained through trapping and evaluating the population dynamic at both the fruit orchard level and the area-wide landscape (comprised of a mix of cultivated, natural and urban areas). Adult medfly is particularly suited to sampling with traps, because of its high mobility and the existence of commercial lures [33].

Based on current knowledge, many factors affect determination of the spatial and temporal dynamics of *C*. *capitata*. These include the leking behaviour of both sexes, the host plants and fruit availability, landscape composition, habitat preferences and the seasonal dispersal of medflies [33–39].

There are relatively few studies that have explicitly analysed the medfly spatial distribution pattern. Those that have, usually involve precision IPM or area-wide SIT programmes, where areas of aggregation are identified as targets for the application of control techniques [33,35,39–42]. Papadopoulos et al. [35] highlighted different aggregation distributions between males and females, suggesting that the two sexes respond in different ways to environmental variables in orchards. However, the spatial distribution of females has never been related to their mating state (unmated or mated), despite the fact they respond to different stimuli [43].

This study was carried out over three years in a peach orchard in central Italy. The aim was to analyse the medfly catches obtained with two trapping systems (using Jackson trap and VasoTrap^®^). Adult sex (male or female) and mating status of females (unmated or mated females) were determined in order to show their spatio-temporal dynamic and the factors affecting the pest distribution in the experimental field.

## Materials and methods

### Study area

**Fig 1:**
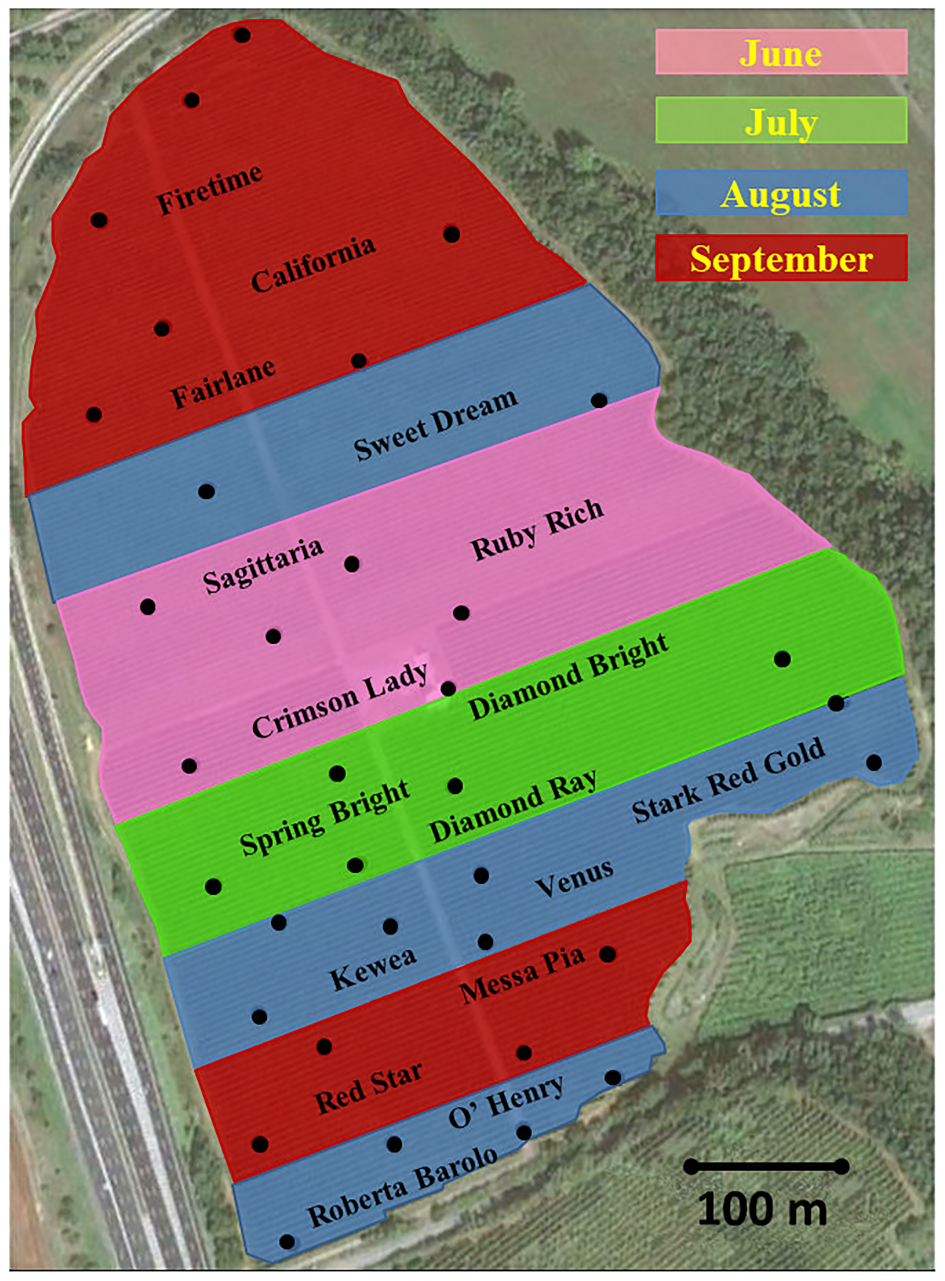
Map of the peach orchard located near Rome, central Italy. The name of the cultivar indicates its position in the orchard. Colours represent the harvesting month of different cultivars. Dots show the position of sampling sites.

The field trials were conducted in a multivarietal peach orchard of approximately 23 hectares located near Rome (central Italy), in a flat agricultural landscape, at N 41°54ʹ22ʺ latitude and E 12°44ʹ06ʺ longitude and 47 m above sea level (Fig 1).

Major roads from northwest to southwest delimited the borders of the orchard, wild vegetation surrounded the northeast perimeter and olive trees and kiwi plants flanked the southeastern side.

The multivarietal peach orchard included 17 different cultivars ripening from the beginning of June to mid-September (Table 1). At the beginning of the survey, the peach trees were around 10 years old, bar the 1-year-old cultivar ‘Sweet Dream’. The planting system was linear and rows were oriented from southwest to northeast. Each cultivar occupied a variable number of rows. Early cultivars (harvested in June) were located in the middle of the orchard and at the northern and southern ends, there were increasingly late cultivars (Fig 1). The area occupied by each cultivar varied from 0.6 to 2.3 hectares (Table 1).

**Table 1:** Area occupied and periods of ripening (grey colour), from veraison to harvest, reported for each cultivar in the orchard.

Cultivar	Area	Veraison and harvest period													
		May		June			July			August			September		
Sagittaria	0.5 ha														
Crimson Lady	2.1 ha														
Diamond Bright	1.4 ha														
Ruby Rich	1.8 ha														
Spring Bright	1.0 ha														
Diamond Ray	1.1 ha														
Sweet Dream	2.1 ha														
Venus	0.9 ha														
Stark Red Gold	1.0 ha														
Roberta Barolo	0.5 ha														
O’ Henry	0.6 ha														
Kewea	0.9 ha														
Red Star	1.0 ha														
California	1.3 ha														
Fairlane	1.4 ha														
Firetime	2.3 ha														
Messa Pia	1.1 ha														

At harvest, all fruit was collected, apart from a few remaining on the trees or on the ground. Other fruit orchards were located in the surrounding areas, mainly peach and kiwi, with no citrus fruit orchards. Over three years (2011–2013), the orchard was subjected to chemical treatments. The number of applications in each cultivar varied year by year depending, amongst other factors, on the maturation period of the fruits. As a result, cultivars were subjected to a number of treatments ranging from 1 to 5. The active ingredients used in rotation were deltamethrin, chlorpyrifos, and etofenprox.

No specific permissions were required for these activities as the research was carried out in a private farm. The owner of the land gave his permission to conduct the study on this site. The field studies did not involve endangered or protected species.

### Data collection

The activity of *C*. *capitata* adults was monitored using a male-targeted trimedlure-baited delta-type Jackson trap (Novapher, San Donato Milanese, Italy) and a female-targeted wet device VasoTrap^®^ (Roberto Carello, Torino, Italy), baited with Biolure^®^ Unipack (Suterra Europe Biocontrol, Gavá, Spain), composed of ammonium acetate, trimethylamine and putrescine (S1 Fig). Trimedlure dispensers were replaced every 4 weeks and the sticky boards every 2–4 weeks. The Biolure Unipack bait was replaced once each season.

The monitoring period lasted three years (2011–2013). Two traps for each peach cultivar were positioned using a grid of geo-referenced sampling points (Fig 1). In 2011 and 2012, 35 sampling points were identified in the peach orchard. One Jackson trap and one VasoTrap were mounted for each point, at 20 m apart, from the end of June and until end of December. In 2013, 30 VasoTraps were set up from June to December. All traps were mounted at 2 meters height on the tree and on the southern side of the raw to minimise variability due to the trap position.

Medflies caught in the traps were removed and counted on a weekly basis. All females were put in tubes containing saline solution and kept at a temperature of -20°C until dissection of the spermathecae. Under stereoscopic microscope, the entire female reproductive tract was removed by pulling the aculeus with a pair of pliers and added to saline solution. The spermatheca was then isolated and transferred in a drop of water to a microscope slide (25 mm × 75 mm) and covered with a 20 mm × 20 mm glass slide that was causing the rupture of the spermathecae. The slide was observed at a 400x magnification to distinguish between unmated (sperm absent) or mated (sperm present) females (S1 Video).

For the collection of climatic data, we referred to the Guidonia weather station (Roma, Italy, latitude N 42°0′0″, longitude E 12°43′0″), 6 km away from the experimental field (http://www.ilmeteo.it/portale/archivio-meteo/Guidonia).

### Data analysis

#### Blind test

Since the mating status of the females (unmated or mated) was inferred based on visual observation of sperm in the spermathecae, a preliminary blind test was carried out in order to assess whether absence of sperm equals virgin status (unmated female) and presence of sperm equals fecundated status (mated female). The blind test was carried out in order to avoid subjective bias by the investigator.

The first investigator collected two types of *C*. *capitata*:

- Unmated females emerged from individually isolated laboratory pupae that had never been in contact with males.
- Mated females, selected only following visual verification in the laboratory of mating with a male (discarding cases of short copulation).

Each medfly was stored individually in a test tube with a physiological saline and labelled with a code that related to the relevant category (unmated or mated) on a blinded sheet. Subsequently, the test tubes were mixed.

A second investigator, absent during the first stage, analysed the spermathecae of all females noting the results for each labelled individual on a second sheet.

The results were compared by means of a contingency table and χ ^2^ analysis with Yates correction for small samples. The overall accuracy of the prediction was calculated as a percentage of observed females correctly assigned to the correct mating status (unmated female = spermathecae without sperms; mated female = spermathecae with sperms) versus females not correctly assigned (unmated females = spermathecae with sperms; mated females = spermathecae without sperms).

#### Statistical analysis

A correlation analysis was carried out, calculating the Pearson correlation coefficient, on the data from the trap catches to assess the relationships between: males trapped with Jackson trap versus males trapped with the VasoTrap; males versus unmated/mated females; and unmated females versus mated females.

To evaluate the effect of the cultivar and chemical treatments on trap catches, a two-way ANOVA was carried out on the total number of males (from Jackson traps and VasoTraps) and unmated and mated females captured, with cultivar, chemical treatment and the factor interaction cultivar x chemical treatment as the main effects. Games-Howell post-hoc test was used to highlight trap catch differences among the cultivars and chemical treatments.

Three levels of chemical treatment were evaluated according to the number of treatments received by the cultivars each year: low chemical input (one treatment at most), medium chemical input (two or three treatments) and high chemical input (four or five treatments).

Catches were defined as the number of flies per trap per day. Normal distribution of the data and homogeneity of the variance were evaluated using the Shapiro-Wilk test and the Levene test, respectively, and data were ln (x+1) transformed to approximate a normal distribution. All statistical analyses were carried out using the statistical software SPSS version 18 (SPSS Inc., Chicago, Illinois, USA).

#### Spatial analysis

Geostatistical methods were used to characterise the spatial distribution of Jackson trap or VasoTrap males, unmated fermales and mated females. The spatial dependence among trap catches was examined by calculating omnidirectional semivariograms with a maximum distance of 380 m, using GS+ version 7 (Gamma Design Software, Plainwell, Michigan, USA).

Experimental variograms were fitted according to the model that gave the lowest residual sum of squares (RSS). Linear, spherical, exponential and Gaussian functions were tested using the software GS+. Models were defined by the nugget (C0), the range (a) and the sill (C). The range allows estimation of the distance at which two points are no longer correlated and, thus, the minimum distance between sampling points that is statistically and spatially independent [44]. The ratio C0:C, known as the k parameter, was used to evaluate the amount of randomness that exists in the data at distances smaller than the sampling distance. Values approaching 1 indicate that the distribution is more aggregated [45]. Data were elaborated for monthly catches, expressed as the mean number of flies per trap per day.

For each year, a one-way ANOVA and Sheffe post-hoc test, were carried out to compare ranges of aggregated distributions obtained from Jackson trap males, VasoTrap males and unmated and mated females. Normal distribution of the data and homogeneity of the variance were evaluated using the Shapiro-Wilk test and the Levene test, respectively, and data were ln (x) transformed to approximate a normal distribution.

Models obtained from semivariograms were used to interpolate data by means of the kriging algorithm using Surfer software version 12 (Golden software, Golden, Colorado, USA) where x and y represented latitude and longitude, expressed as Universal Transversal Mercator (UTM) coordinates, and z represented the trap counts. The interpolation output was a grid file showing values in a regular, rectangular array.

To compare distributions obtained from different sets of data, regardless the number of catches, sampling data in the grid file were normalised as follows:

$$n_{i\_norm}=\frac{n_{i}}{n_{max}}$$ (1)

where n_i_norm_ represents the normalised value of *n*_*i*_; *n*_*i*_ is the i-esim value of z and *n*_*max*_ is the maximum value of z in the same dataset. This was realised using the ‘grid/math’ option in Surfer to obtain a new interpolation grid file for each considered dataset, with the same spatial pattern of the untransformed one and normalised values ranging between 0 and 1.

The grid obtained was used to produce the contour map, which showed the configuration of the surface by means of isolines representing equal z-values. A base map showing the orchard, with the same coordinate system, was placed on top of the contour map. Zones of the contour map with higher values than surrounding areas were referred to as ‘hot spots’. Contour maps were elaborated monthly through each experimental year.

## Results

### Blind test

The first investigator selected 108 females, 43 of them unmated and 65 mated. The second investigator, who carried out the dissection and detection of the sperm in the female spermathecae, detected 44 females without sperms (unmated) and 64 females with sperm (mated).

The overall accuracy was 99.1%. The χ ^2^ test showed a statistically significant result (χ^2^ = 99.88; df = 1; P<0.0001) and confirmed the reliability of the spermathecal analysis to assess the female mating status.

All females collected in the traps were then submitted to the dissection and were classified as unmated or mated females.

### Medfly captures

During the sampling period (2011–2013), 72,749 medfly adults were collected in all traps in the peach orchard. In the Jackson traps, only males were observed, with a sporadic number of females counted (these were not considered in the following statistical analyses). The number of specimens in the Jackson traps were 14,775 and 15,573 in 2011 and 2012, respectively. In the VasoTraps, 42,401 specimens were collected, 45% males and 55% female, and of the latter, 59% were unmated and 41% were mated. In 2011, 6,759 adults were collected (50% males, 35.4% unmated females and 14.6% mated females). In 2012, 24,365 specimens were collected (40.4% males, 35.2% unmated females and 24.4% mated females). In 2013, 11,277 specimens were collected (51.6% males, 25.1% unmated females and 23.3% mated females).

Correlation analysis of males obtained from Jackson traps and males obtained from VasoTraps was highly significant for both 2011 and 2012, with r indicating a positive relationship between the Jackson traps and VasoTraps (Table 2).

**Table 2:** Results of the correlation analysis carried out to assess the relationships between males obtained by VasoTrap and Jackson trap; total males with unmated and mated females; and unmated females with mated females.

Comparison	year	r
**VasoTrap males vs. Jackson trap males**	2011	0.844*
	2012	0.865*
**Unmated females vs. total males**	2011	0.799*
	2012	0.827*
	2013	0.892*
**Mated females vs. total males**	2011	0.607*
	2012	0.458*
	2013	0.570*
**Mated females vs. unmated females**	2011	0.691*
	2012	0.674*
	2013	0.472*

* P<0.001

Correlation analyses to compare males with females, although always significant, gave different results. The relationship between males and unmated females was high for all three years (Table 2). The relationship between males and mated females was lower, with r ranging between 0.458 in 2012 and 0.607 in 2011 (Table 2).

The correlation analyses between unmated and mated females (Table 2) gave variable correlation in the three years from low in 2013 (r = 0.472), to medium in 2011 and 2012 (r between 0.674 and 0.691) (Table 2).

### Temporal dynamic

**Fig 2:**
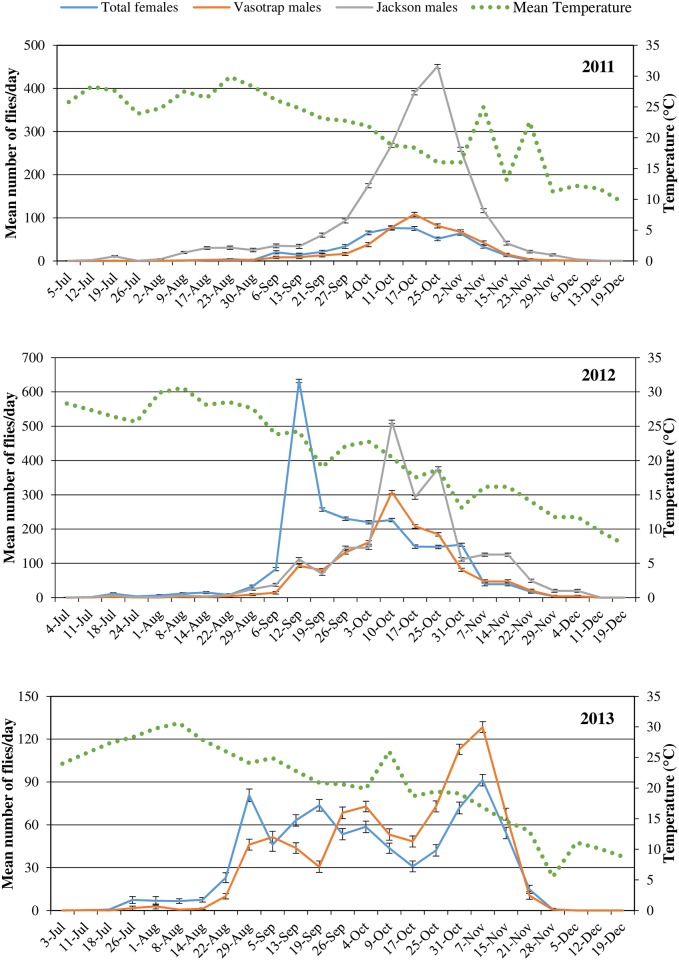
Weekly *Ceratitis capitata* catches of females and males trapped in VasoTraps and males trapped in Jackson traps, expressed as the mean (±SE) number of flies per trap per week in 2011, 2012 and 2013. Average weekly data for mean temperature were obtained from daily recordings from the meteorological station located near the peach orchard.

**Fig 3:**
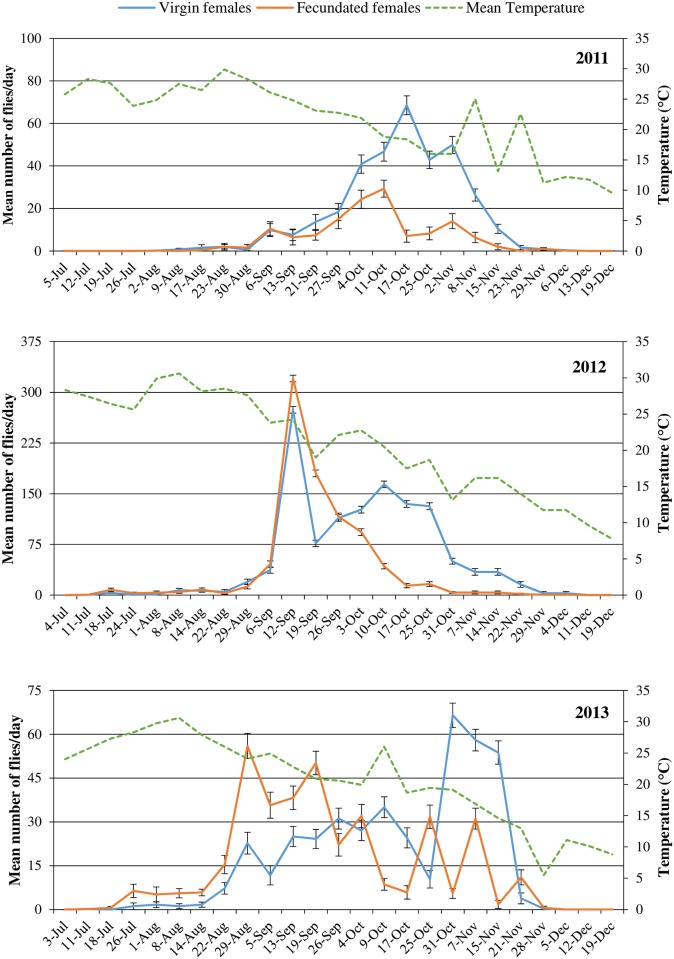
Weekly *Ceratitis capitata* catches of unmated and mated females, expressed as the mean (±SE) number of flies per trap per week in 2011, 2012 and 2013. Average weekly data for mean temperature were obtained from daily recordings from the meteorological station located near the peach orchard.

In 2011, the first adult capture was recorded on 5th July. The number captured slightly increased in August and increased more abruptly in September to the beginning of December. The major peak for males was in October (Fig 2), and was higher in the Jackson traps than the VasoTraps. The number of females started to increase in the second half of August, with clear differences between unmated and mated individuals: the first ones had a peak in mid-October, when the number of mated females fell sharply (Fig 3). The first capture was obtained from a Jackson trap, followed 2 weeks later by both males and females (unmated and mated) in the VasoTraps.

In 2012, the first capture occurred at the beginning of July and remained at low level until September, when they increased abruptly, peaking at the beginning of September and mid-October for females and males, respectively (Fig 2). Catches ended at the beginning of December. Jackson traps had a higher number of catches compared to VasoTraps in October but not in the other months. Unmated and mated females showed a similar trend until a peak in September. In the following weeks, the unmated females showed a new peak in October, that was not observed in mated females (Fig 3). The first capture was again obtained in a Jackson trap, followed after one week by both males and females (unmated and mated) in the VasoTraps.

In 2013, the first individuals were trapped at the beginning of July. For both sexes captures increased in mid August and showed three peaks in the following months; captures were terminated at the end of November. Females had a peak at the end of August, mid-September and the beginning of November, whereas males showed a one-week delay in the first two peaks and a coinciding third peak (Fig 2). Mated females were captured more frequently than unmated females until the end of September; after which a peak of unmated females was observed at the end of October (Fig 3).

### Cultivar and chemical treatment effects

The peach cultivars, chemical treatments and their interaction significantly influenced the trap catches in all the ANOVA analyses. Results were analysed separately according to sex and reproductive status (unmated females and mated females) (Table 3).

**Table 3:** Results of ANOVA on the effect of cultivar and chemical treatments in male and unmated and mated female captures.

Dependent variable	Source of variation	d.f.	Mean squares	F	P
Total males	Treatment	2	3.082	3.565	0.029
	Cultivar	16	4.804	5.556	<0.001
	Treatment*Cultivar	8	3.558	4.115	<0.001
	Error	1884	0.865	-	-
Unmated females	Treatment	2	0.119	0.347	0.707
	Cultivar	16	2.306	6.738	<0.001
	Treatment*Cultivar	8	2.107	6.157	<0.001
	Error	1722	0.342	-	-
Mated females	Treatment	2	1.228	4.609	0.010
	Cultivar	16	1.029	3.862	<0.001
	Treatment*Cultivar	8	1.246	4.676	<0.001
	Error	1722	0.267	-	-

**Fig 4:**
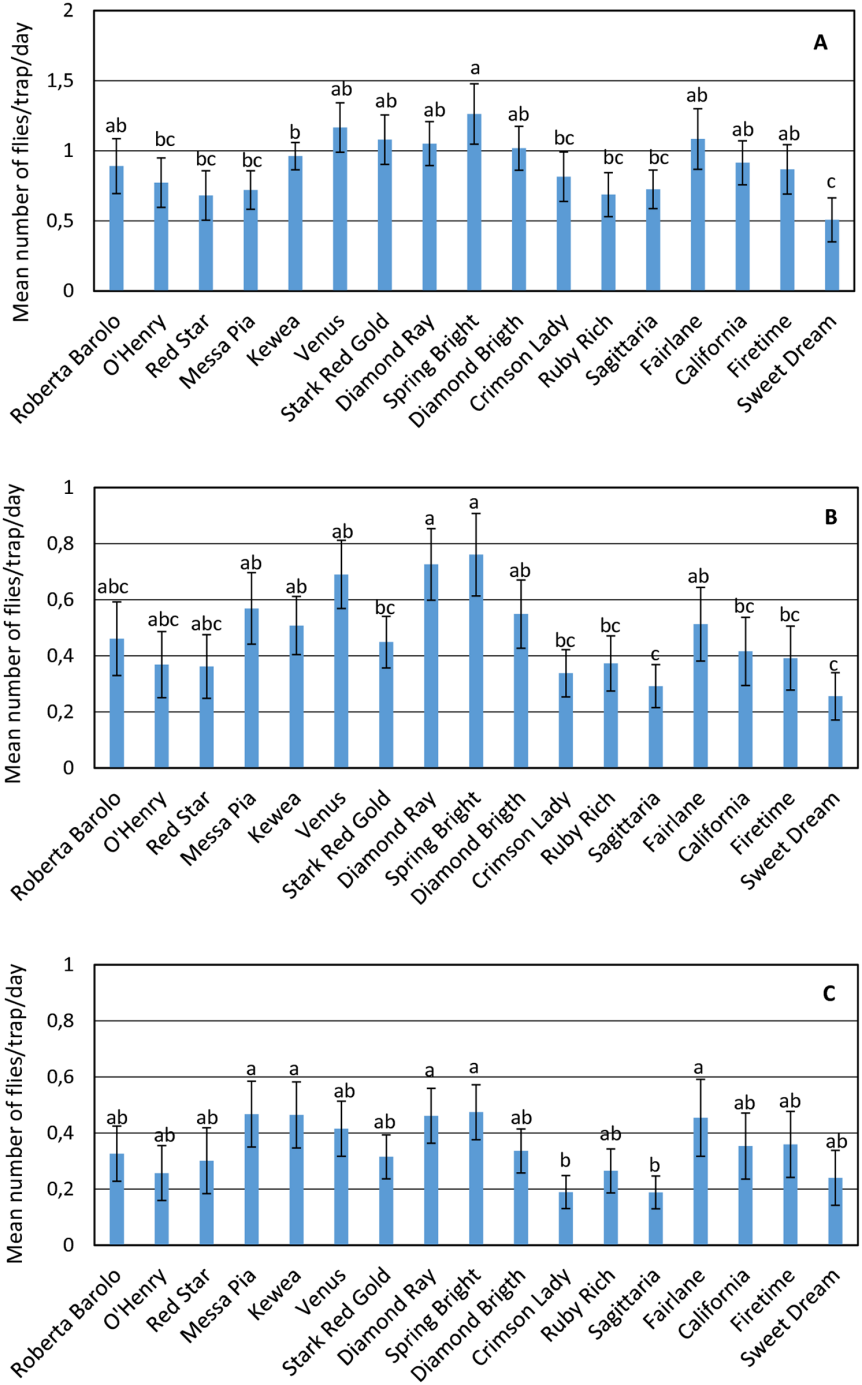
Mean (±SE) number of flies per trap per day obtained from the 3-year sampling trap catches collected in peach cultivars in the orchard. Calculated for total males (A), unmated females (B) and mated females (C).

The ANOVA carried out for males showed that ‘Spring Bright’, harvested at the end of July, had the highest number of catches; the lowest numbers were observed in ‘Sweet Dream’, with fruit collected at the beginning of August (Fig 4A). The effect of chemical treatments on male trap catches was significant. However, a post-hoc test for chemical treatment did not show differences between groups with different chemical treatments.

For unmated females, ‘Spring Bright’ and ‘Diamond Ray’, both harvested in July, caught the highest number of flies; ‘Sagittaria’ and ‘Sweet Dream’, harvested in June and August, respectively, had the lowest number (Fig 4B). Chemical treatment was not significant, although the interaction with the cultivar was.

Mated females differed significantly among cultivars and chemical treatments. Two mid cultivars (‘Diamond Ray’ and ‘Spring Bright’) and three late cultivars (‘Messa Pia’, ‘Kewea’ and ‘Fairlane’) showed the highest number of captures, whereas ‘Crimson Lady’ and ‘Sagittaria’, both with early harvesting times (June), showed the lowest number of trapped individuals (Fig 4C). A significantly higher number of catches was observed in the group that received high chemical treatment compared with the mid and low treatment groups.

Interactions were also significant for all dependent variables tested, indicating a combined effect of cultivar and chemical treatment. Additionally, the same chemical treatment affected capture levels differently for different cultivars. Consider ‘Diamond Ray’, male captures significantly increased from low chemical treatment groups, whereas for ‘Sagittaria’ male captures significantly decreased. ‘Red Star’ captures significantly increased from mid to high chemical treatment. In other cultivars, such as ‘Venus’, no variations were observed shifting from low to high chemical treatment. Analogous interactions were observed for unmated and mated female catches, but showed variations in the single cultivars from those observed in males.

### Spatial analysis

In 2011, all models elaborated from experimental semivariograms had an asymptotic function, with the exception of December for Jackson trap males and July and October for unmated females (S1 Table). The most common model is spherical (in 70% of cases). The range varied from 44 m to 2,432 m, with an average of 403±110 m. The k parameter indicates strongly aggregated distributions (> 0.75) in 87% of cases. ANOVA comparing ranges obtained from different variables was not significant (df: 3,19; F = 0.688; P = 0.57).

**Fig 5:**
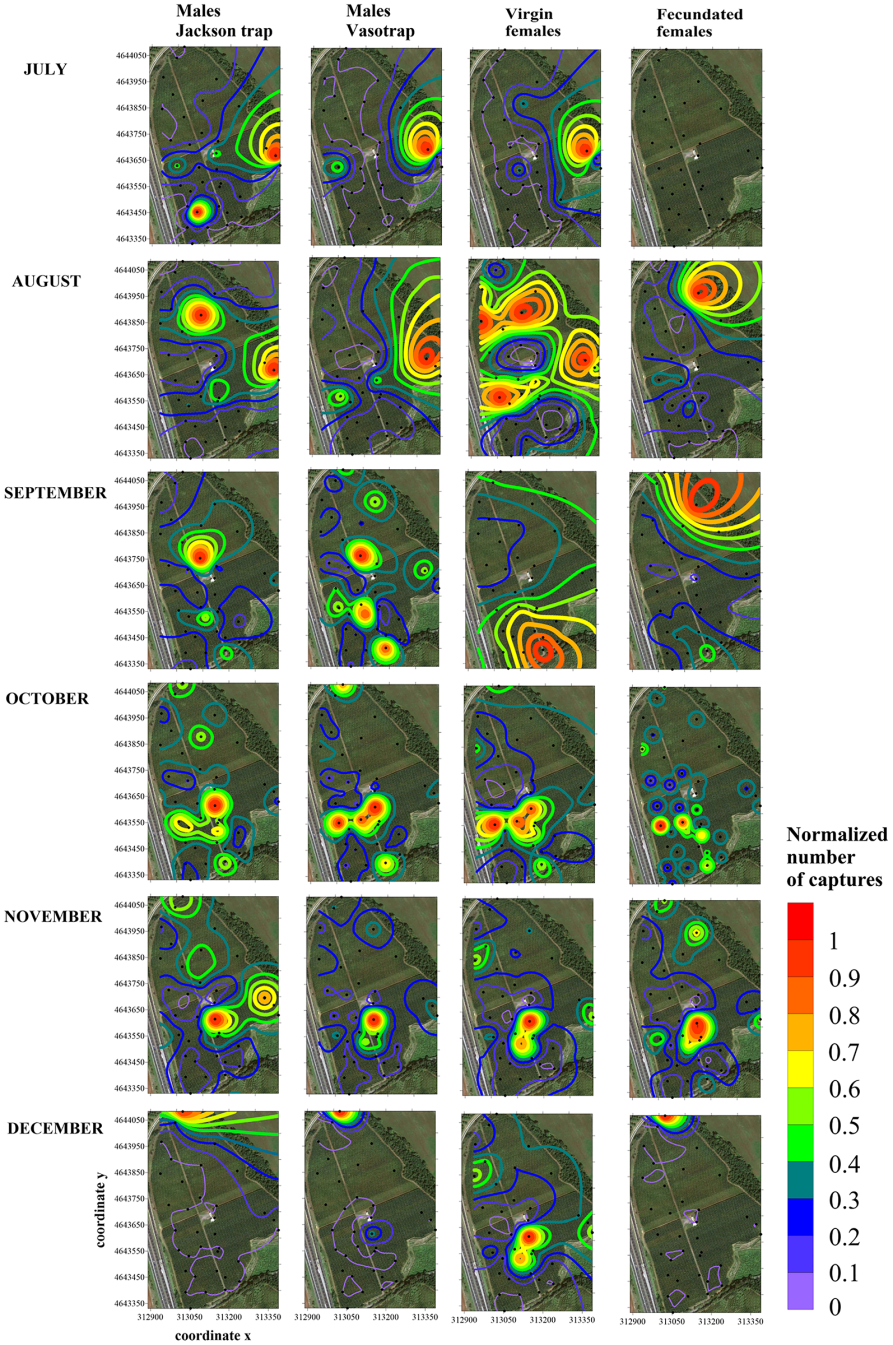
Contour maps showing the medfly hot spot distribution obtained using the kriging algorithm applied to 2011 monthly trap counts of males (from Jackson traps and VasoTraps) and unmated and mated females. Trap locations in the field are represented by black dots; x and y axes are expressed in UTM coordinates.

The maps show the hot spot distribution clearly varying with time and for each considered variable (Fig 5).

In July, both males and females were localised in the middle of the peach orchard toward the northeastern border, corresponding to the ripening of fruit on cultivars ‘Diamond Ray’ and ‘Diamond Bright’. Jackson trap males showed a small hot spot around ‘Messa Pia’, a mid-harvest cultivar. During this month, no unmated females were collected.

In August, the hot spot observed in July continued, but both Jackson trap males and unmated females had an additional hot spot (extended for unmated females) localised on late cultivar ‘Fairlane’. Mated females showed a different distribution, most of them being concentrated on the late cultivar ‘California’, adjacent to the ‘Fairlane’ cultivar.

In September, both Jackson trap and VasoTrap males had strong aggregated distributions mostly on cultivars with fruit already harvested, with the latter showing some additional minor hot spots. Unmated and mated females showed very different distributions: unmated females were captured in the southern zone of the orchards on cultivar ‘Roberta Barolo’, whose fruits were collected in mid-August, in part overlapping with the male distribution; mated females were mostly aggregated in the northern part of the orchard on the cultivar ‘California’, as in August.

In October and November, when no fruits were on the trees, males and females aggregated in the middle of the orchard on the adjoining early cultivars ‘Spring Bright’, ‘Stark Red Gold’ and ‘Kewea’ (in November) and, on the southern border, ‘Roberta Barolo’ (in October). Distributions tended to overlap, with mated females more dispersed in the field than males and unmated females. In November, small hot spots appeared in the northern part of the orchard. In December, the position of the last catches coincided for males and mated females on the northern border of the orchard (late cultivar ‘Firetime’). Conversely, unmated females aggregated on the same cultivars observed in November.

In 2012, the variogram analysis indicated an aggregated spatial distribution, with spherical function in all cases (S2 Table). The range varied from 113 to 811 m, with an average of 445±65 m. The k parameter indicated strongly aggregated distributions (> 0.75) in 85% of cases. ANOVA comparing ranges of different variables was not significant (df: 3,16; F = 0.134; P = 0.94).

**Fig 6:**
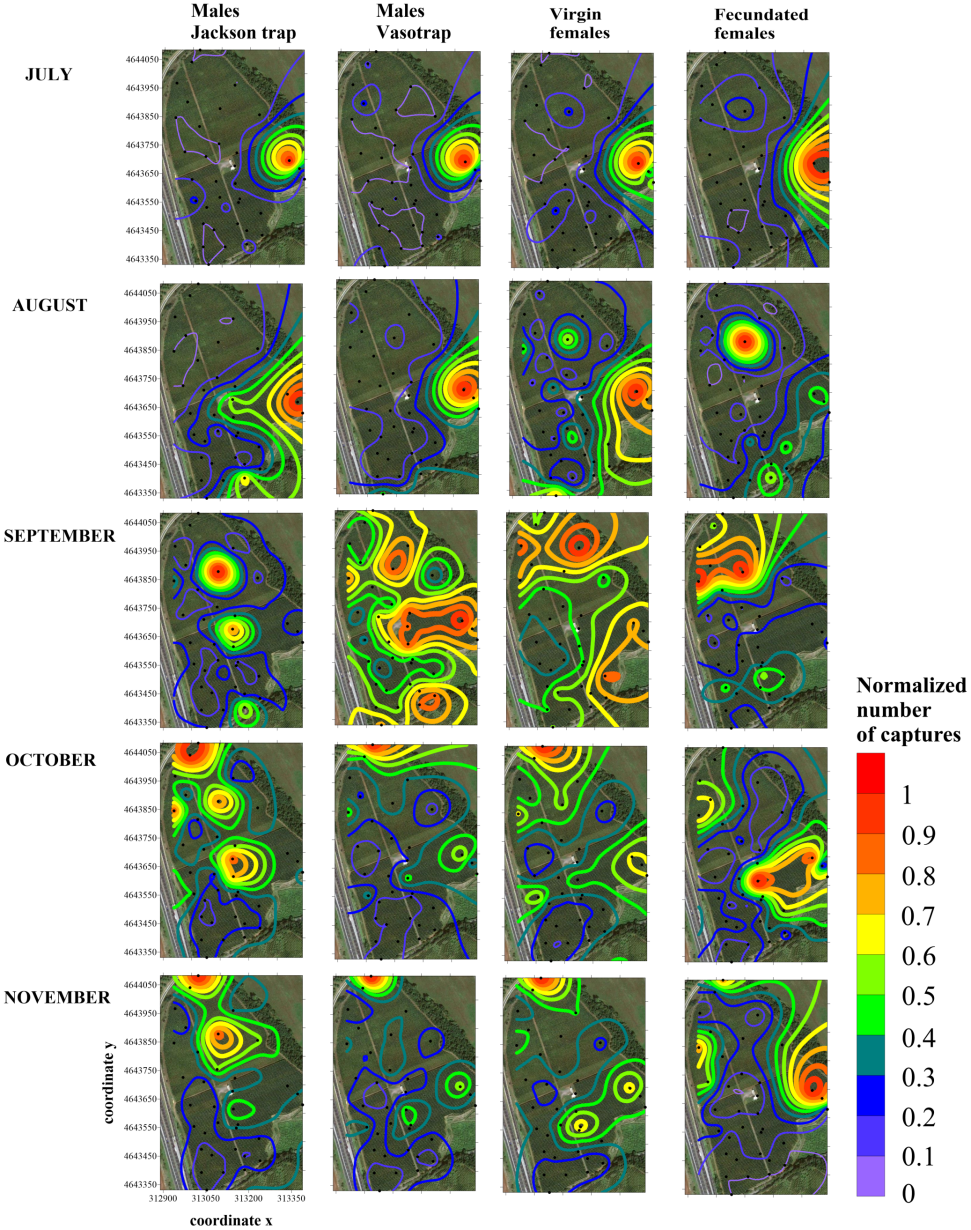
Contour maps showing the medfly hot spot distribution obtained using the kriging algorithm applied to 2012 monthly trap counts of males (from Jackson traps and VasoTraps) and unmated and mated females. Trap locations in the field are represented by black dots and x and y axes are expressed in UTM coordinates.

**Fig 7:**
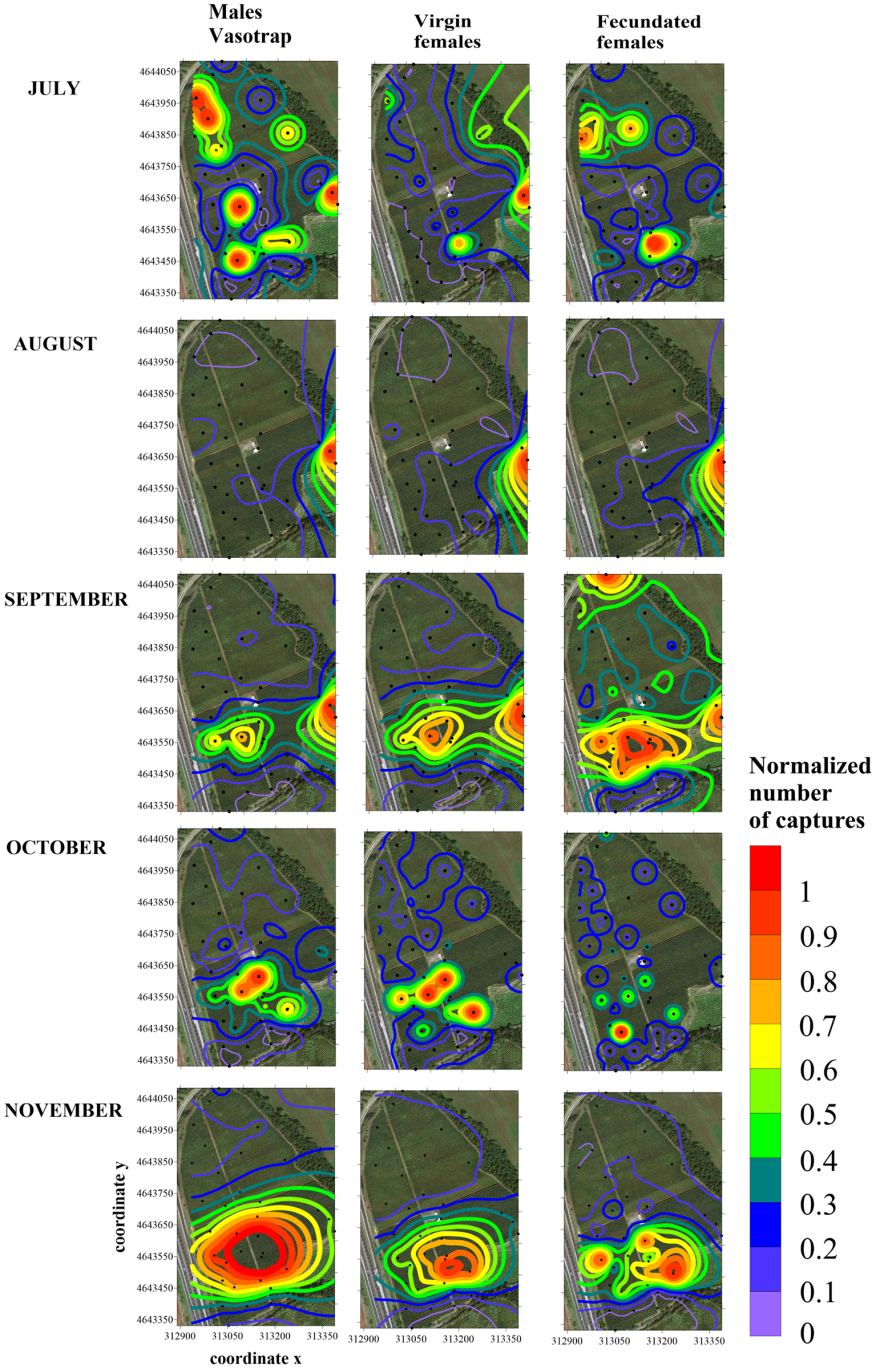
Contour maps showing the medfly hot spot distribution obtained using the kriging algorithm applied to 2013 monthly trap counts of males and unmated and mated females. Trap locations in the field are represented by black dots and x and y axes are expressed in UTM coordinates.

The distributions of the first catches (in July) were similar for all the medflies, and were the same of those observed in 2011 (Fig 6). In August, the main hot spot did not change for males and unmated females, with only small differences, when the harvesting time for those cultivars was completed. In contrast, mated females captures shifted to a different zone, corresponding to the cultivar ‘Fairline’, with additional minor hot spots visible along the southeastern border. In September, Jackson traps showed three strongly aggregated hot spots, on cultivars ‘Roberta Barolo’, ‘Crimson Lady’ and ‘Fairline’. The same distribution was seen for VasoTrap males, although more dispersed. Unmated females occupied a large area in the northern and southern areas of the orchard, whereas mated female distribution remained stable, with a hot spot on cultivar ‘Fairline’ expanding to adjoining cultivars. In October and November, the distribution started to aggregate on the northern border with the disappearance of the other hot spots and a spatial pattern very similar to the Jackson trap males, VasoTrap males and unmated females. The mated females followed different dynamics, with the main hot spot located on the eastern border, corresponding to the early cultivars ‘Diamond Ray’ and ‘Diamond Bright’, the same area as the first mated females collected in August 2011 and 2013 (Figs 5 and 7).

In 2013, all models elaborated from the semivariograms had an asymptotic function, except in July for unmated females (S3 Table). The most common model was spherical (in 93.3% of cases). The range varied from 49 meters to 811 m, with an average of 338±72 m. The k parameter indicated strongly aggregated distributions (> 0.75) in 87% of cases. The ANOVA comparing ranges of different medfly variables was not significant (df = 2,12; F = 0.571; P = 0.58).

The distribution of first few individuals in July had a scattered distribution in the orchard, but in August, one single hot spot was observed for all three medfly categories (Fig 7), coincident with the last mated female distribution in 2012 (Fig 6).

In the following three months (September to November), the distribution of males and unmated females was similar and mainly aggregated in areas of the orchard with early cultivars, extending to adjoining mid and late cultivars in October and November. Mated females showed some differences, particularly in September, when the distribution extended to cultivars with fruit, such as ‘Firetime’ and ‘Messa Pia’, or recently harvested fruit, such as ‘Kewea’, ‘Venus’ and ‘Stark Red Gold’.

## Discussion

During the three-years sampling period, *C*. *capitata* adult catches occurred from the first half of July to the beginning of December. Abundance varied depending on the time of year between late August and early November. The observed dynamic was in agreement with the phenology of the medfly in temperate zones and monocultural orchards, in which the host plant fruits were not available for certain periods of the year [39,46].

The temporal trend of male captures in the two trap types (Jackson trap and VasoTrap) was very similar, but the Jackson trap started to capture 1–2 weeks earlier and more individuals were collected during the sampling period. There were also differences in the male spatial patterns, particularly the lack of or low intensity hot spots in the VasoTrap, at different times over two years (July-to August 2011 and September to November 2012). These results were not unexpected. The Jackson trap, baited with trimedlure, has been widely used as a male medfly attractant [47–49]. Males showed a minor attraction towards food-based attractant contained in VasoTrap, than females [50]. When the two traps are placed together, the Jackson trap has a much higher number of trapped males than the VasoTrap. From a practical perspective, the Jackson trap with trimedlure best represented the spatio-temporal dynamic of the male medfly, particularly in the early part of the season with low population levels, leading to effective insecticide interventions. Not taking into consideration females, the information provided by the Jackson trap is limited, for example in relation to the distribution of mated females or the potential risk of damage to the fruit. Consequently, in precision IPM programmes it is preferable to use traps baited with food attractants such as biolure.

The comparison between males and females highlights a number of differences. At the beginning of the seasonal flight, the number of females captured was higher than the number of males, but this is later reversed and overall the males become overwhelming with respect to the females. Interestingly, the unmated females regularly increase during the season, becoming more abundant of mated females in the last sampling period. The observed dynamic can be explained by a decrease in temperature that reduces the number of medfly mating [46,51], the paucity of fruits to oviposit, the dispersion of mated females [35], and the length of life of unmated females [52]. The number of males and unmated females captured in the traps showed good correlation, whereas the correlation of males or unmated females with mated females was low. Similar results have also been shown in spatial patterns, as discussed below.

The spatial analysis of data, from both variograms and contour maps, clearly showed aggregated distributions. A small number of random distributions can be traced back to the first seasonal catches, when populations were low, or to late months when no fruit was available and the medflies tended to have a random distribution in the orchard [35,39]. The calculated semivariogram ranges, which represent the size of an aggregation area (hot spot), did not show significant differences between trap type, sex or mating status of females. These results are consistent with that reported by Meats and Smallridge [7] in a mark recapture trial, who showed that 90% of the flies remain within a 400–700 m radius from the point of release, although the behaviour of released flies might be different from that of feral ones.

In contrast, the location of hot spots in the orchard during the sampling seasons showed great differences between sex and female mating status. As medfly adults were free to move in the orchard, the spatial distribution observed on the contour maps is a result of the natural dispersal of the population. This is affected by endogenous and exogenous stimuli acting on single individuals at different moment of their lifetime. Aggregations of males in hot spots overlapped with unmated females and were in different locations to aggregations of mated females. There were significant differences in the distribution of unmated females and mated females for almost all months in 2011 and 2012, whereas male and unmated female distributions overlapped in July, August, October and November.

The first hot spots in the season (July or August) were located on the eastern side of the orchard, with bordering a wooded area. At the end of the season (November or December), the most common hot spot ocation was in the north corner of the orchard where later cultivars were found. During these periods, the distributions of males and unmated females and mated females tended to coincide more so than in other months. It can be inferred from studies by Papadopoulos et al. [53] and Sciarretta and Trematerra [39], that *C*. *capitata* adults overwinter in wild host plants neighbouring the orchard using preferential ecological corridors to enter them. However, the maps themselves cannot clarify this.

Variations in catches on different cultivars, though significant, were not constant over the three-year sampling period and, for males, were less apparent. Mated females showed variations based on fruit maturation period, with more catches in late cultivars observed.

Similar results were shown for the relationship between peach cultivars and hot spot positions in the distributional maps. Males and unmated and mated females tended to share hot spots when fruit was still available over many cultivars, and male and unmated female distribution tended to diverge from mated females when few late cultivars remained and males aggregated on cultivars without fruits.

Differences also emerged when different levels of chemical treatment were considered. In the case of males and unmated females no differences were observed, however, large numbers of mated females were caught in cultivars subjected to high levels of chemical treatment. This last observation may appear to be contradictory, but mated females were most attracted to late cultivars that would have received the highest amount of chemical treatment. In addition, the significant interaction effects in the results of ANOVA indicate non-homogeneous variations in the captures from the same cultivar at different levels of chemical treatment. Importantly, the spatial distribution of medfly seems not to be affected by chemical treatments, most likely due to their natural dispersal in the field, that hidden the killing effect of pesticides. This result has important practical implications as it suggests that for control measures to be effective, area-wide IPM principles should be taken into account. This will prevent the omission of other medfly location, for example areas of shelter.

Previous studies have identified environmental factors that affect the spatio-temporal distribution, including landscape structure, host plant location and fruit ripening sequence of host plants [35,39]. Our results demonstrated that endogenous factors can be equally significant, showing differentiated patterns and dynamics related to medfly sex and female mating status. These differences are due to the diverse sources of attraction, which depend on the physiological states of each individual [54].

Males aggregated at feeding sites, where they acquire food sources and volatile compounds like α-copaene for signalling activities, and leking sites, usually the tree canopy, where they find shelters and optimal microhabitats for mating [34,55]. Areas of aggregation often form on host plants, because of the increased possibility of contact with females searching for food and oviposition sites [56], however, the factors influencing male aggregation sites were not clarified in our study, as opposed to the unmated and mated females. Our results showed that male distribution was poorly linked to the presence of fruit on trees, and in fact, males often aggregated on cultivars without fruits, where no chemical treatments had taken place.

Unmated females, two—three days after emerging, are attracted to the male pheromone over fruit. They visit the leks, where males aggregate and compete to defend their small territory in which they court females [43,57–59]. As a result of such behaviour, unmated females had a spatial distribution pattern that largely overlapped with males (through not exactly coincident most likely due to the increased demand for proteins by females) [50]. After mating, females become attracted to host fruits for oviposition [43,50] and the distribution of mated females becomes closely related to the phenology of host plant. In the case of this study, the mated female distribution is related to the sequence of fruit ripening in the different peach cultivars, and causes a divergence from the male and unmated female distributions. New stimuli explain the higher number of mated females around late cultivars, where fruit is on the tree for longer, despite the increased treatment with pesticides. The distributional maps of mated females consistently reflects this searching behaviour, with hot spots mainly located on cultivars with fruits, and completely absent from early cultivars (June to July), where fruit was collected before or at the beginning of the medfly annual seasonal flight.

Results gave relevant insights into pest monitoring and Integrated Pest Management (IPM). The location of male hot spots (and consequently of unmated females) around peach cultivars without fruit demonstrated that male aggregations are unrelated to plant phenology. Thus, it would be risky to omit monitoring and control from these parts of the orchard or host plants because there is no fruit on the trees, and the hot spot position must be continuously monitored [60].

Traps with male attractants such as trimedlure generally gave a better picture of medfly spatio-temporal dynamics than Biolure, but do not catch mated females, that are responsible for larval infestations. As several food-based attractants have been investigated in recent years [61–63], we believe an important challenge will be to specifically assess the monitoring of mated females. In fact, treating females as a whole can produce unreliable results, as unmated females and mated females have very different spatial distribution patterns in some periods.

However, in the light of the results shown here, locating male hot spots through monitoring allows localisation of virgin female populations. This finding will be important in the application of IPM programs. In fact, pest control directed at areas of male aggregation will also affect the virgin female populations, and will consequently lower the population of fecundated females. This hypothesis can be further tested by comparing a field subjected to whole-area chemical application, with a hotspot-treated field, observing the fruit damage levels at harvest, to verify if a precision IPM that includes target males, and therefore unmated females, coupled with effective sanitation practices, is an effective approach to medfly control.

## Supporting information

S1 Fig VasoTrap, baited with Biolure Unipack and positioned on a peach tree for adult medfly sampling in the peach orchard. (JPG)

S1 Table Models and parameters calculated from the experimental semivariograms obtained in 2011 from monthly trap catches of Jackson trap males, VasoTrap males, unmated females and mated females. (DOCX)

S2 Table Models and parameters calculated from the experimental semivariograms obtained in 2012 from monthly trap catches of Jackson trap males, VasoTrap males, unmated females and mated females. (DOCX)

S3 Table Models and pa^r^ameters calculated from the experimental semivariograms obtained in 2013 from monthly trap catches of VasoTrap males, unmated females and mated females. (DOCX)

S1 Video Video showing sperm contained in the spermathecae of several mated females under a microscope. (MP4)
