# Supplementary figures and images for: Analysis of the Mediterranean fruit fly [Ceratitis capitata (Wiedemann)] spatio-temporal distribution in relation to sex and female mating status for precision IPM
Source: PLoS One. 2018 Apr 4;13(4):e0195097. doi: 10.1371/journal.pone.0195097 (PMC5884526; doi:10.1371/journal.pone.0195097)

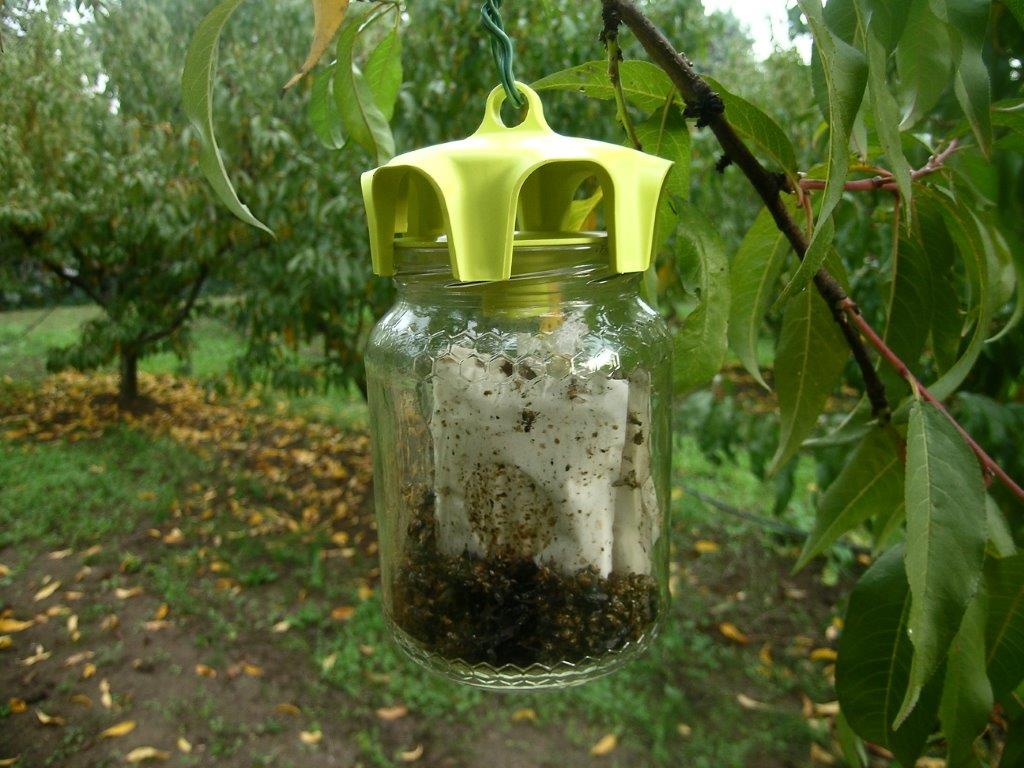

Supplement: S1 Fig — (JPG) [file pone.0195097.s001.jpg]
