# Supplementary material for: Analysis of the Mediterranean fruit fly [Ceratitis capitata (Wiedemann)] spatio-temporal distribution in relation to sex and female mating status for precision IPM
Source: PLoS One. 2018 Apr 4;13(4):e0195097. doi: 10.1371/journal.pone.0195097 (PMC5884526; doi:10.1371/journal.pone.0195097)
Supplement: S1 Table — (DOCX) [file pone.0195097.s002.docx]

**S1 Table.** Models and parameters calculated by the experimental semivariograms obtained in 2011 from monthly trap catches of the following variables: Jackson trap males, VasoTrap males, unmated females and mated females.

| **Months** | **Model** | **Nugget** | **Sill** | **Range** | **RSS** | **r^2^** | **K** |
| --- | --- | --- | --- | --- | --- | --- | --- |
| Jackson trap males | | | | | | | |
| July | exponential | 0.060 | 0.160 | 2432 | 8.8x10^-4^ | 0.45 | 0.84 |
| August | spherical | 0.001 | 1.261 | 306 | 0.06 | 0.93 | 1.00 |
| September | exponential | 0.001 | 2.116 | 267 | 0.16 | 0.87 | 1.00 |
| October | spherical | 4.200 | 38.180 | 87 | 84.70 | 0.20 | 0.89 |
| November | spherical | 0.001 | 1.852 | 162 | 0.08 | 0.95 | 1.00 |
| December | linear | 0.010 | 0.013 | 314 | 1.4x10^-4^ | 0.03 | 0.24 |
| VasoTrap males | | | | | | | |
| July | spherical | 0 | <0.001 | 558 | 1.4x10^-9^ | 0.94 | 1.00 |
| August | spherical | 0.001 | 0.013 | 529 | 1.8E-6 | 0.96 | 0.97 |
| September | spherical | <0.001 | 0.102 | 88 | 1.6x10^-3^ | 0.52 | 1.00 |
| October | spherical | 0.280 | 4.460 | 79 | 7.82 | 0.52 | 0.94 |
| November | spherical | 0.055 | 0.347 | 79 | 0.03 | 0.053 | 0.84 |
| December | spherical | 0 | <0.001 | 132 | 4.5x10^-8^ | 0.63 | 1.00 |
| Unmated females | | | | | | | |
| July | linear | <0.001 | <0.001 | 319 | 2.0x10^-8^ | 0.80 | 0.87 |
| August | spherical | <0.001 | 0.001 | 213 | 5.9x10^-7^ | 0.57 | 0.92 |
| September | spherical | 0.041 | 0.111 | 503 | 1.5x10^-4^ | 0.92 | 0.63 |
| October | linear | 2.021 | 2.021 | 290 | 1.15 | 0.00 | 0.00 |
| November | spherical | 0 | 0.152 | 97 | 0.07 | 0.40 | 1.00 |
| December | exponential | <0.001 | <0.001 | 1333 | 1.1x10^-8^ | 0.30 | 0.87 |
| Mated females | | | | | | | |
| August | exponential | <0.001 | 0.002 | 405 | 1.4x10^-7^ | 0.87 | 0.96 |
| September | spherical | 0.008 | 0.122 | 782 | 8.1x10^-4^ | 0.95 | 0.94 |
| October | spherical | 0.023 | 0.198 | 44 | 0.01 | 0.00 | 0.88 |
| November | spherical | 0.001 | 0.007 | 95 | 1.3x10^-5^ | 0.19 | 0.93 |
| December | spherical | 0 | <0.001 | 155 | 2.6x10^-10^ | 0.68 | 0.99 |
