# Supplementary material for: Analysis of the Mediterranean fruit fly [Ceratitis capitata (Wiedemann)] spatio-temporal distribution in relation to sex and female mating status for precision IPM
Source: PLoS One. 2018 Apr 4;13(4):e0195097. doi: 10.1371/journal.pone.0195097 (PMC5884526; doi:10.1371/journal.pone.0195097)
Supplement: S2 Table — (DOCX) [file pone.0195097.s003.docx]

**S2 Table.** Models and parameters calculated by the experimental semivariograms obtained in 2012 from monthly trap catches of the following variables: Jackson trap males, Vasotrap males, virgin females and fecundated females.

| **Moths** | **Model** | | **Nugget** | **Sill** | **Range** | **RSS** | **r^2^** | **k** |
| --- | --- | --- | --- | --- | --- | --- | --- | --- |
| Jackson trap males | | | | | | | | |
| July | | spherical | <0.001 | 0.060 | 811 | 2.69x10^-4^ | 0.84 | 1.00 |
| August | | spherical | <0.001 | 0.315 | 811 | 0 | 0.91 | 1.00 |
| September | | spherical | 0.010 | 9.510 | 113 | 2.09 | 0.89 | 1.00 |
| October | | spherical | 0.100 | 45.120 | 182 | 54.50 | 0.96 | 1.00 |
| November | | spherical | 0.784 | 3.114 | 180 | 0.64 | 0.68 | 0.75 |
| VasoTrap males | | | | | | | | |
| July | | spherical | <0.001 | 0.006 | 811 | 3.9x10^-6^ | 0.85 | 1.00 |
| August | | spherical | <0.001 | 0.063 | 811 | 8.2x10^-5^ | 0.96 | 1.00 |
| September | | spherical | 0.243 | 1.780 | 168 | 0.09 | 0.90 | 0.86 |
| October | | spherical | 1.930 | 28.450 | 386 | 107.00 | 0.80 | 0.93 |
| November | | spherical | 0.055 | 0.322 | 135 | 3.1x10^-3^ | 0.85 | 0.83 |
| Unmated females | | | | | | | | |
| July | | spherical | <0.001 | 0.014 | 811 | 1.4x10^-5^ | 0.88 | 1.00 |
| August | | spherical | <0.001 | 0.061 | 413 | 3.5x10^-4^ | 0.86 | 1.00 |
| September | | spherical | 1.170 | 4.628 | 416 | 0.76 | 0.88 | 0.75 |
| October | | spherical | 1.270 | 6.477 | 241 | 4.01 | 0.80 | 0.80 |
| November | | spherical | 0.092 | 0.214 | 168 | 0.00 | 0.65 | 0.57 |
| Mated females | | | | | | | | |
| July | | spherical | <0.001 | 0.064 | 811 | 6.9x10^-4^ | 0.92 | 1.00 |
| August | | spherical | <0.001 | 0.055 | 185 | 1.1x10^-4^ | 0.93 | 1.00 |
| September | | spherical | 0.040 | 10.089 | 386 | 1.4 | 0.97 | 1.00 |
| October | | spherical | 0.039 | 0.292 | 244 | 0.02 | 0.72 | 0.87 |
| November | | spherical | <0.001 | 0.016 | 811 | 1.9x10^-6^ | 0.94 | 0.98 |
