# Supplementary material for: Analysis of the Mediterranean fruit fly [Ceratitis capitata (Wiedemann)] spatio-temporal distribution in relation to sex and female mating status for precision IPM
Source: PLoS One. 2018 Apr 4;13(4):e0195097. doi: 10.1371/journal.pone.0195097 (PMC5884526; doi:10.1371/journal.pone.0195097)
Supplement: S3 Table — (DOCX) [file pone.0195097.s004.docx]

**S3 Table.** Models and parameters calculated by the experimental semivariograms obtained in 2013 from monthly trap catches of the following variables: Vasotrap males, unmated females and mated females.

| **Months** | **Model** | **Nugget** | **Sill** | **Range** | **RSS** | **r^2^** | **K** |
| --- | --- | --- | --- | --- | --- | --- | --- |
| Vasotrap males | | | | | | | |
| July | spherical | <0.001 | 0.001 | 92 | 6.6x10^-8^ | 0.44 | 1.00 |
| August | spherical | 0.001 | 1.816 | 811 | 0.69 | 0.68 | 1.00 |
| September | spherical | 0.010 | 3.613 | 302 | 1.37 | 0.88 | 1.00 |
| October | spherical | 0.010 | 6.181 | 127 | 0.99 | 0.90 | 1.00 |
| November | spherical | 1.660 | 5.498 | 711 | 0.85 | 0.79 | 0.70 |
| Unmated females | | | | | | | |
| July | linear | <0.001 | 0.001 | 314 | 1.7x10^-7^ | 0.49 | 0.52 |
| August | spherical | 0.001 | 0.388 | 811 | 0.02 | 0.70 | 1.00 |
| September | spherical | 0.093 | 0.628 | 289 | 8.3x10^-3^ | 0.95 | 0.85 |
| October | spherical | 0.001 | 1.222 | 80 | 0.08 | 0.50 | 1.00 |
| November | spherical | 0.251 | 1.238 | 294 | 0.10 | 0.85 | 0.80 |
| Mated females | | | | | | | |
| July | spherical | <0.001 | 0.006 | 98 | 5.23 | 0.69 | 0.98 |
| August | spherical | <0.001 | 2.011 | 707 | 0.90 | 0.76 | 1.00 |
| September | spherical | 0.066 | 0.598 | 152 | 2.1x10^-3^ | 0.97 | 0.89 |
| October | spherical | 0.001 | 0.141 | 49 | 7.9x10^-4^ | 0 | 1.00 |
| November | spherical | 0.002 | 0.175 | 233 | 6.8x10^-3^ | 0.74 | 0.99 |
